# Supplementary figures and images for: Acute Myeloid Leukemia Causes Serious and Partially Irreversible Changes in Secretomes of Bone Marrow Multipotent Mesenchymal Stromal Cells
Source: Int J Mol Sci. 2023 May 18;24(10):8953. doi: 10.3390/ijms24108953 (PMC10219446; doi:10.3390/ijms24108953)

## Slide 1
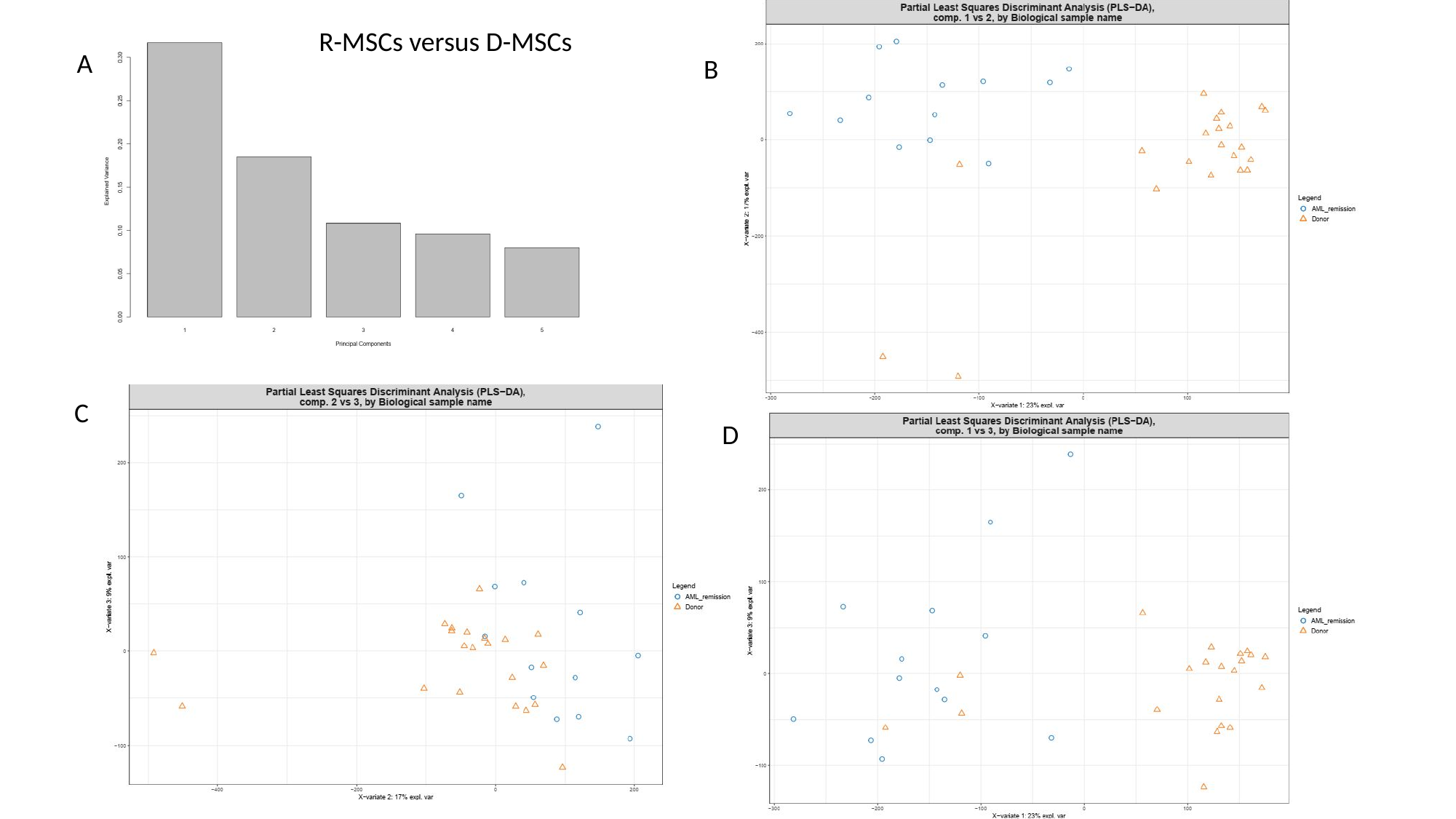

R-MSCs versus D-MSCs
A
B
C
D

Supplement: Supplementary file 1 [file ijms-24-08953-s001.zip › Supplement Figure S1.pptx]

## Slide 1
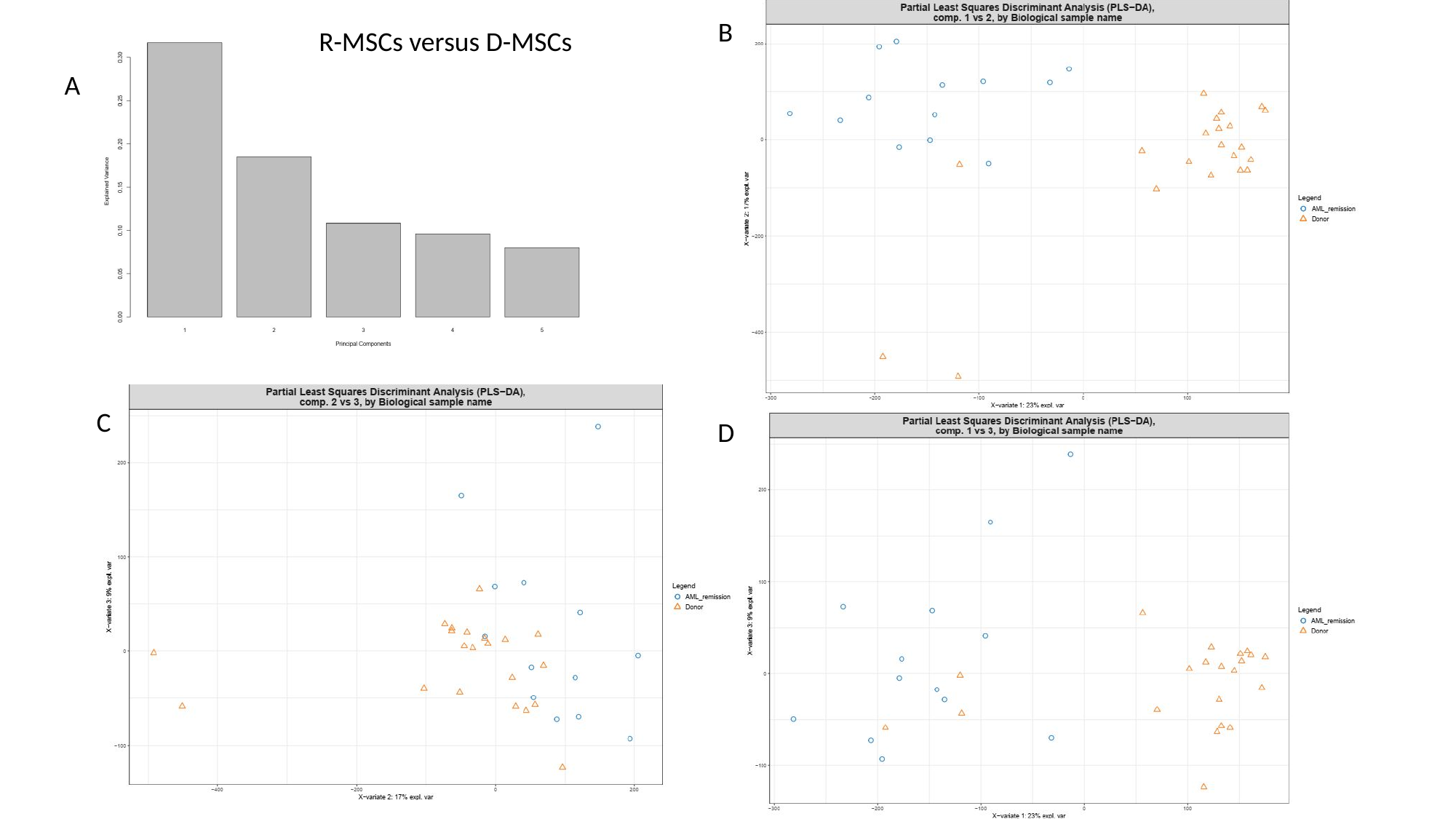

B
R-MSCs versus D-MSCs
A
C
D

Supplement: Supplementary file 1 [file ijms-24-08953-s001.zip › Supplement Figure S2.pptx]

## Slide 1
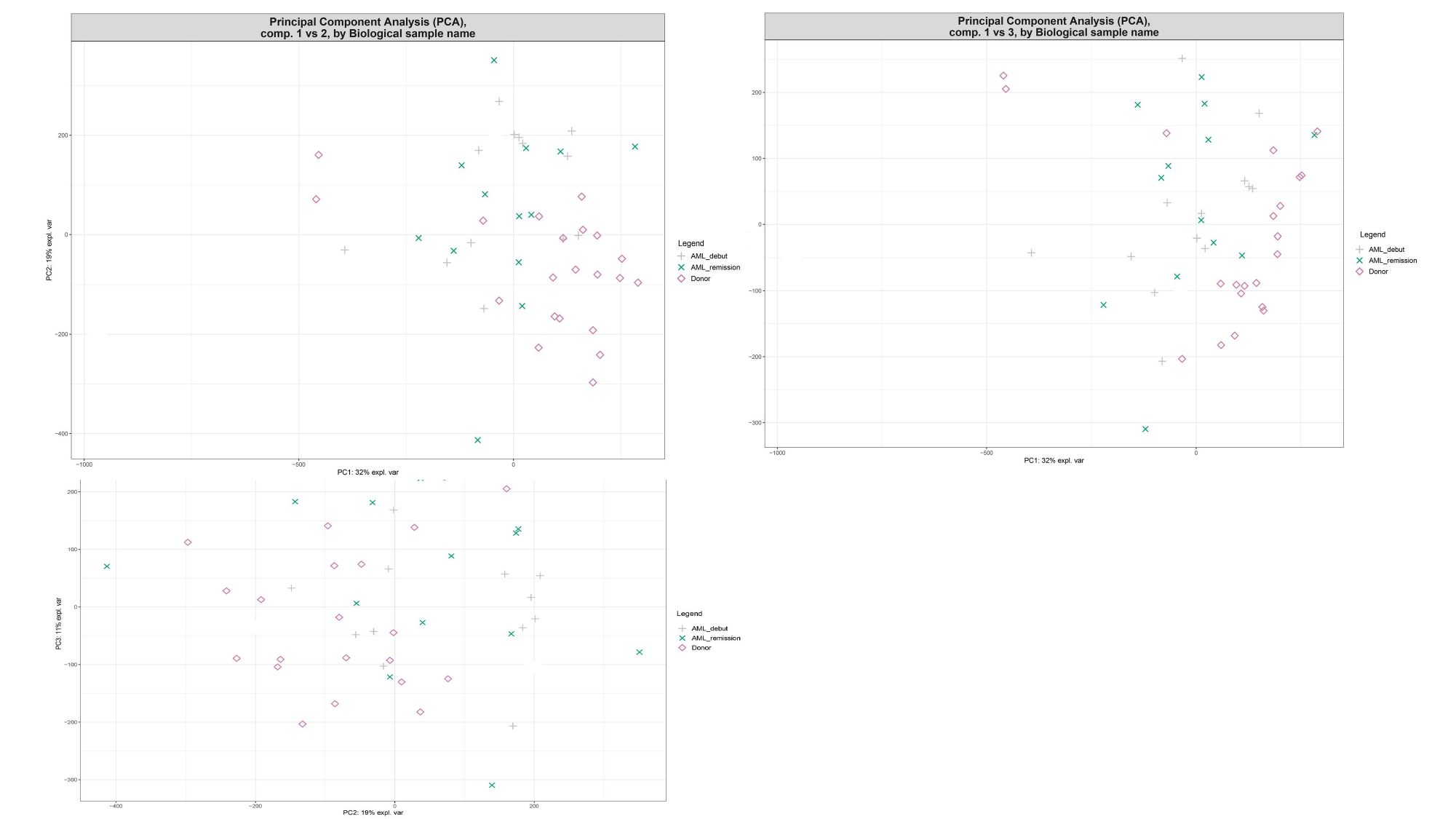

Supplement: Supplementary file 1 [file ijms-24-08953-s001.zip › Supplement Figure S3.pptx]
